# Clinical characteristics in subjects with *NLRP3 *V198M diagnosed at a single UK center and a review of the literature

**DOI:** 10.1186/ar4171

**Published:** 2013-02-19

**Authors:** Dorota M Rowczenio, Hadija Trojer, Tonia Russell, Anna Baginska, Thirusha Lane, Nicola M Stewart, Julian D Gillmore, Philip N Hawkins, Patricia Woo, Bozena Mikoluc, Helen J Lachmann

**Affiliations:** 1 National Amyloidosis Centre, Centre for Amyloidosis and Acute Phase Proteins, Division of Medicine Royal Free Campus, UCL Medical School, Rowland Hill Street, London NW3 2PF, UK; 2 Division of Infection & Immunity, UCL, Rayne Building, 5 University Street, London WC1E 6JF, UK; 3 Department of Pediatrics and Developmental Disorders, Children's Teaching Hospital, Waszyngtona Street 17, Bialystok 15-224, Poland

## Abstract

**Introduction:**

Mutations in the *NLRP3 *gene are associated with the dominantly inherited cryopyrin-associated periodic syndrome (CAPS). The significance of the V198M variant is unclear; it has been reported in association with various CAPS phenotypes and as a variant of uncertain consequence. The aim of this study was to characterize the clinical phenotypes and treatments in individuals with V198M assessed in a single UK center.

**Methods:**

DNA samples from 830 subjects with fever syndromes or a family history of CAPS were screened for mutations in the *NLRP3 *gene with polymerase chain reaction (PCR) and sequencing. A detailed medical history was available in all cases. Inflammatory disease activity was monitored monthly with measurements of serum amyloid A protein (SAA) and C-reactive protein (CRP) in symptomatic individuals.

**Results:**

*NLRP3 *V198M was identified in 19 subjects. It was found in association with CAPS in five cases, in one patient with Schnitzler syndrome, in three patients who also had a nucleotide alteration in another fever gene, and in three other patients with evidence of an autoinflammatory phenotype. Seven asymptomatic individuals were detected during screening of family members.

**Conclusions:**

The *NLRP3 *V198M variant shows variable expressivity and reduced penetrance. It may be associated with classical inherited or apparently sporadic CAPS and with atypical autoinflammatory disease of varying severity, intriguingly including Schnitzler syndrome. The factors that influence the pathogenic consequences of this variant remain unknown. However, the remarkable response to interleukin 1 (IL-1) blockade in all but one individual in our series confirms that their clinical features are indeed mediated by IL-1.

## Introduction

Cryopyrin-associated periodic syndrome (CAPS) is a rare autoinflammatory disorder associated with overproduction of IL-1β [1]. CAPS encompasses three individual phenotypically overlapping syndromes of differing severity, originally described as distinct entities but all subsequently linked to mutations in a single gene [2-5]. Familial cold autoinflammatory syndrome (FCAS, MIM 120100) was first described by Kile and Rusk in 1940 [6] and is the least severe, characterized by recurrent episodes of fever, urticaria-like rash, arthralgia, and inflammatory eye manifestations induced by exposure to cool or damp environment [7,8]. Muckle-Wells syndrome (MWS, MIM 191900) resembles FCAS, but the disease is more severe and persistent and less obviously influenced by ambient temperature. Systemic inflammation in MWS starts in infancy or early childhood [9], and variable degrees of sensorineural deafness, often beginning in adolescence, occur in more than 60% of cases. Without effective treatment, between a quarter to a third eventually develop systemic AA amyloidosis [10]. Chronic infantile neurologic cutaneous articular syndrome (CINCA, MIM 607115), also known as neonatal-onset multisystem inflammatory disease (NOMID), has the most severe phenotype and presents in the neonatal period with involvement of many organs, including the skin, the skeleton, and the central nervous system. Dominant inheritance is evident in about 75% of patients with MWS and FCAS, whereas CINCA is usually sporadic; *de novo *mutations in *NLRP3 *can be identified in about half of patients with CINCA, but the presumed genetic etiology in the remainder is yet to be characterized [5,11]. Symptomatic CAPS is accompanied by a striking acute-phase response, with serum concentrations of C-reactive protein (CRP) and serum amyloid A protein (SAA) frequently elevated by 100- to 1,000-fold, underlying the high risk of AA amyloidosis [10,12].

To date, 138 sequence variants of *NLRP3 *have been identified, of which 110 have been associated with CAPS, and 28 are either nonpathogenic or of undetermined significance [13], the commonest of which is the Q703K variant (also described as Q705K in older literature) [14] that has been reported in 5% of healthy Caucasian alleles [11]. Pathogenic *NLRP3 *variants result in substantial upregulation of caspase 1-mediated cleavage of pro IL-1β and secretion of active IL-1β, although the precise molecular mechanisms by which this occurs are yet to be elucidated fully. The *NLRP3 *V198M variant (previously described as V200M) [10] is of particular interest because it has been reported in both individual patients and families with apparently classical CAPS [4,10,15]; in association with other autoinflammatory syndromes [16,17]; in patients with apparently acquired autoinflammatory disorders including the IgM paraprotein-related Schnitzler syndrome [18]; and in healthy Caucasian controls with an allele frequency of between 0.0038 [10] and 0.0074 [11].

We describe here the clinical phenotypes and treatments in individuals with V198M characterized in a single UK center and review the published literature regarding the clinical spectrum associated with this variant.

## Materials and methods

### Subjects and relatives

The *NLRP3 *gene was analyzed in 830 subjects assessed at the single UK specialist center between 2002 and 2011. The subjects comprised patients with suspected autoinflammatory disease (90%) and a family history of CAPS, including family members of individuals in whom the V198M was identified (10%). Informed consent was provided by all subjects, and the ethical approval for the study was obtained from Royal Free Hospital and University College Medical School Research Ethics Committee for this retrospective study (REC reference number 06/Q0501/42)

### Methods

#### Genetic analysis

DNA was extracted as previously described [19]. Exon 3 of *NLRP3/CIAS1 *[NCBI RefSeqGene (LRG_197)] was amplified in three fragments with the following primers: nucleotides: g.12654 to g.13428 with forward: 5'-GTTACCACTCGCTTCCGATG-3' and reverse: 5'-CCTCGTTCTCCTGAATCAGAC-3'; nucleotides: g.13397 to g.13972 with forward: 5'-CATGTGGAGATCCTGGGTTT-3' and reverse: GGCCAAAGAGGAAACGTACA-3' nucleotides: g.13955 to g.14523 with forward: 5'-ACTACCTGCTGGAAGAGGAA-3' and reverse: 5'-GCTGTGGCAACAGTATTTGGA-3'.

Negative and positive controls were included in each run. PCR was validated with gel electrophoresis, and PCR products were purified with a QIAquick PCR purification kit (Qiagen, Velno, The Netherlands) according to the manufacturer's protocol. Sequencing reaction was performed with Big Dye Terminator v. 3.1 Ready Reaction Cycle Sequencing kit (Applied Biosystems, Warrington, UK). The electrophoretic profiles of *NLRP3 *sequences were analyzed on the ABI 3130xl Genetic Analyser by using Sequencing Analysis Software version 5.4.

Subjects who displayed features that may be seen in more than one of the currently described autoinflammatory syndromes underwent additional mutation screening of the following genes: *MEFV *(the gene associated with familial Mediterranean fever (FMF)) exons 2 and 10; *TNFRSF1A *(the gene associated with TNF receptor-associated periodic syndrome (TRAPS)) exons 2 to 7 including introns 2, 4, and 6; and *MVK (*the gene associated with mevalonate kinase deficiency (MKD), also known as hyper-IgD and periodic fever syndrome (HIDS)) exons 9 and 11.

#### Serial measurements of the acute-phase proteins

Serial measurements of the acute-phase proteins SAA and CRP were monitored monthly in symptomatic individuals. SAA was measured in serum with latex nephelometry (BNII autoanalyzer; Dade Behring, Marburg, Germany) [20]. The lower limit of detection was 0.7 mg/L, with an interassay coefficient of variation (CV) of 2.6% at 15 mg/L and 3.7% at 80 mg/L. CRP was measured in serum by using a high-sensitivity automated microparticle-enhanced latex turbidimetric immunoassay (COBAS MIRA; Roche Diagnostics GmbH) [21,22]. The lower limit of detection was 0.2 mg/L, with an interassay CV of 4.2% at 4 mg/L and 6.3% at 1 mg/L.

## Results

### Genetic analysis

Pathogenic *NLRP3 *variants were identified in 78 (9%) of the 830 screened subjects; most frequent was R260W, found in 23 (29%) cases, and V198M, identified in 19 (24%) individuals. We have not identified a second *NLRP3 *variant, including the low-penetrance polymorphisms in the 19 subjects with V198M, but three had an amino acid variation in another fever gene: one was a compound heterozygote for *MVK *H44fs/V377I; one was heterozygous for a novel variant in *TNFRSF1A *Y38S, and one was heterozygous for *MEFV *E148Q. The low-penetrance variants Q703K and R488K were found in 59 (7.1%) and four (0.5%) of the 830 screened cases, respectively.

### Phenotypes associated with V198M

Clinical features associated with *NLRP3 *V198M and response to treatment are summarized in Table 1.

**Table 1:** Phenotypes associated with *NLRP3 *V198M variant and response to treatment

Kindred/subjects	Syndrome	Age at presentation (years)	Median SAA Pre/post treatment	Median CRP Pre/post treatment	Treatment	Mutations in other genes: *MEFV, TNFRSF1A*, and *MVK*
Family 1/Subject 1	CAPS (MWS/FCAS/CINCA)	Neonatal	193/3	42/1	Anakinra CR	None

Family 1/Subject 2	CAPS (MWS/FCAS/CINCA)	Neonatal	121/2	56.5/1	Anakinra CR	None

Family 1/Subject 3	CAPS (MWS/FCAS/CINCA)	Neonatal	133/3	42/1	Anakinra CR	None

Family 1/Subjects 13 and 14	Asymptomatic carriers	-	8	4	ND	ND

Family 2/Subject 4	CAPS (FCAS-type features)	11	ND / 3	ND / 1	Canakinumab CR	None

Family 2/Subjects 15 and 16	Asymptomatic carriers	-	3	1	ND	ND

None/Subject 5	CAPS (MWS-type features)	3	52/ND	ND	ND	None

Family 3/Subject 6	Schnitzler syndrome	57	299/6	156/1	Anakinra CR	None

Family 3/Subjects 17 and 18	Asymptomatic carriers	-	2	2	ND	ND

None/Subject 7	Positive rheumatoid factor polyarthritis	16	174 Lost to F/U	20 Lost to F/U	Anti-TNF therapy CR	None

None/Subject 8	Inflammatory syndrome	9	88/ND	16/ND	ND	None

None/Subject 9	Inflammatory syndrome	6 months	ND	133/ND	ND	None

Family 4/Subject 10	Undiagnosed chronic inflammatory syndrome	7	91.5/NR to treatment	54/NR to treatment	Anakinra NR	*MEFV *E148Q

Family 4/Subject 19	Asymptomatic carrier	-	3	2	ND	ND

None/Subject 11	MKD	Before 10	70/2	21/11	Anakinra PR	*MVK *H44fs/V377I

None/Subject 12	TRAPS complicated by AA amyloidosis	3	236.5/4	53.5/4	Anakinra CR	*TNFRSF1A *Y38S

CR, complete response; ND, not done; NR, no response; PR, partial response.

### Patients with CAPS phenotype

**Figure 1:**
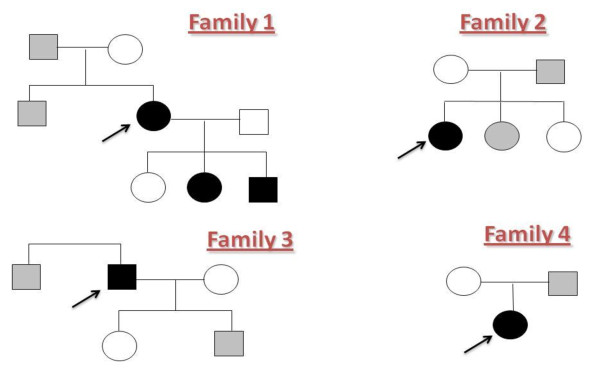
**Pedigrees of four families with *NLRP3 *V198M**. Family 1 with three members affected by MWS/FCAS/CINCA, family 2 with a proband diagnosed with familial cold autoinflammatory syndrome (FCAS), family 3 with a proband affected by Schnitzler syndrome and family 4 with a proband affected by uncharacterized long-standing systemic inflammatory disorder. Open shapes represent healthy individuals, solid shapes represent affected individuals, and shaded shapes represent asymptomatic carriers; each proband is indicated by an arrow.

The V198M variant was identified in a Caucasian family with Muckle-Wells syndrome but with features overlapping both with FCAS and CINCA/NOMID (Family 1). The proband (subject 1) presented neonatally with a widespread urticarial rash affecting the entire body, particularly the trunk and distal limbs. The rash occurred on a daily basis and worsened with stress or after exposure to damp or cold. Other symptoms included swollen joints and severe ankle pain, generalized arthralgia, and hearing impairment, which was first noticed at age 5 years. Because of progressive hearing loss, hearing aids were required from the age of 27. Two of the proband's children (subjects 2 and 3) have the same syndrome, and both developed sensory neural deafness in late childhood. All three subjects displayed mildly dysmorphic features characteristic of CINCA/NOMID, including short stature, frontal bossing of the skull, and flattening of the nasal bridge. The one unaffected child does not carry the V198M variant (Figure 1).

**Figure 2:**
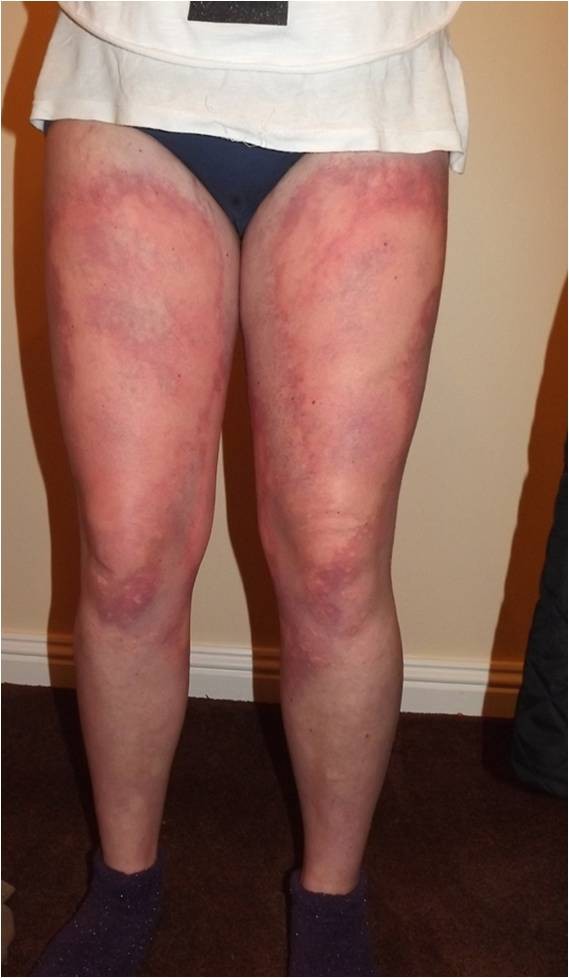
**Diffuse rash precipitated by cold or damp environment in subject 4 diagnosed with familial cold autoinflammatory syndrome (FCAS)**.

V198M was associated with FCAS in a Caucasian subject with no family history of CAPS (Figure 1), who developed a very diffuse urticarial rash in late childhood (subject 4, family 2). The rash was clearly precipitated by exposure to cold or damp environment and was associated with severe limb aching and swelling of the hands, face, legs, and feet (Figure 2). No ocular inflammation, hearing loss, or headaches were noted.

A diagnosis of MWS was made in a child of Northern European ancestry in association with V198M (subject 5), who presented at age 2 years with recurrent fever attacks lasting 2 to 14 days accompanied by arthralgia, myalgia, a diffuse urticarial rash, and headaches. The hearing was unaffected, and the febrile attacks were not precipitated by exposure to cold or damp environment.

### Patient with Schnitzler syndrome

V198M was an unexpected finding in a Caucasian patient who developed persistent flulike symptoms in the sixth decade accompanied by urticarial rash, general malaise, headaches, myalgia, night sweats, and mouth ulcers (subject 6, family 3). These inflammatory symptoms were clearly precipitated by stress. Initially thought to have vasculitis, for which extensive investigations had been made, the patient had failed to respond to broad-spectrum immunosuppression, rituximab, or anti-tumor necrosis factor (TNF) therapy. After the detection of a low-grade IgM κ-secreting lymphoplasmacytic lymphoma, the diagnosis of Schnitzler syndrome was made (Figure 1).

### Patients with other autoinflammatory phenotypes

V198M variant was found in a subject of Arabic ancestry, first seen at the age of 16 years with an aggressive positive rheumatoid factor polyarthritis (subject 7).. There was a partial response to methotrexate but the patient then developed 2.6 g/day proteinuria. A renal biopsy was apparently normal, showing no evidence of AA amyloid deposits, and anti-TNF therapy resulted in a good response with resolution of proteinuria.

V198M was identified in two children; one of Caucasian ancestry who had 2- to 3-day attacks of fever accompanied by cervical lymphadenopathy, myalgia, headache, and abdominal pain; the other was Asian and presented with fever and rash at the age of 6 months (subjects 8 and 9).

### Patients with compound genetic variants

We identified three subjects who, in addition to V198M, also had an amino acid variation in another fever syndrome.

First was a Caucasian subject with an uncharacterized long-standing systemic inflammatory disorder with prominent bony abnormalities (subject 10, family 4), first seen at the age of 7 years with lower-leg and knee pain and was initially thought to have systemic onset juvenile idiopathic arthritis (SoJIA). An osteotomy of the left femur was performed in the early teens, followed by extensive reactive bone growth with a honeycomb appearance on radiograph above the resection site. After developing similar hyperosteotic appearances at the distal end of the right femur SAPHO (synovitis, acne, pustulosis, hyperostosis, and osteitis) syndrome was considered, although none of the other features was present. The patient never experienced episodes of fever or serositis, and the single episode of rash was a drug reaction after antibiotics during the late teens. Screening of the genes associated with other hereditary autoinflammatory syndromes, FMF, TRAPS, and MKD, revealed a heterozygous status for the *MEFV *E148Q polymorphism.

The other two subjects were first seen before the age of 10 years (subject 11) and in the eighth decade with AA amyloidosis and a life-long history of fevers and episodic abdominal pain (subject 12). The first case was diagnosed with two *MVK *variants, H44fs and V377I, and met the clinical criteria for diagnosis of MKD. The second patient was found to be heterozygous for a novel variant in *TNFRSF1A *Y38S, and the inflammatory syndrome was thought to be fully consistent with TRAPS.

### Asymptomatic carriers of V198M

V198M appeared to have no clinical consequences in seven asymptomatic carriers detected as part of family screening: (subjects 13 through 19). CRP and SAA measurements in these individuals did not reveal any evidence of subclinical inflammatory disease.

### Laboratory and clinical response to IL-1 therapy

SAA and CRP were measured in 10 of the 12 affected individuals before treatment was commenced and are outlined in Table 1. Median values were SAA 141.5 mg/L (range, 52 to 299 mg/L) and CRP 54, mg/L (range, 20 to 156 mg/L). Eight patients were treated with anti-IL-1 therapy: anakinra in seven including three non-CAPS patients diagnosed with Schnitzler syndrome, TRAPS, and MKD, respectively (at a dose of 100 mg daily for adults and 1 mg/kg for children), and canakinumab in one (at a dose of 150 mg), and all but one (subject 10) responded completely with rapid resolution of symptoms and normalization of SAA concentration to healthy values of <4 mg/L. Subject 7 had been treated with anti-TNF therapy and showed significant improvement.

## Discussion

Cryopyrin-associated periodic syndrome is an inherited autoinflammatory disorder characterized by episodes of fever, urticarial rash, arthralgia, myalgia, eye inflammation, and, in its more severe forms, bony abnormalities and CNS inflammation.

CAPS is caused by autosomal-dominant gain-of-function mutations of the *NLRP3 *gene (NCBI RefSeqGene (LRG_197)) located on chromosome 1q44, resulting in increased production of IL-1β [23-25]. Despite the dramatic advances in unraveling the pathophysiology of CAPS, including identification of the gene [26-28], the NLRP3 inflammasome [1], and the introduction of highly effective treatment in the form of IL-1-blocking drugs [29-31], our understanding of this disease is incomplete. The exact mechanisms by which *NLRP3 *mutations lead to activation of the inflammasome and overproduction of IL-1β remain unclear. At present, an apparent lack of correlation exists between genotype and phenotype in general and even within a family, and the relation between mutation and clinical phenotype can differ markedly between individuals [10]. Both *NLRP3 *R260W and V198M have been reported in patients with phenotypes overlapping FCAS and MWS [3,27,32], and D303N has been associated with MWS and CINCA/NOMID [33]. Forty percent of children with CINCA/NOMID are reported to be "mutation negative" by conventional genomic sequencing from whole blood. Interestingly, recent publications described a high incidence of somatic mosaicism among mutation-negative CINCA patients. Tanaka *et al*. [34] identified somatic mutations in 18 (69.2%) of 26 patients, with the level of mosaicism ranging from 4.2% to 35.8% by subcloning of the PCR products followed by capillary DNA sequencing of more than 100 subclones for each patient [34]. The latest advances in molecular biology allow rapid and accurate diagnoses of mosaic patients by massive parallel DNA sequencing, which can detect base substitutions at an allele frequency as low as 1% with 99.9% confidence [35].

*NLRP3 *V198M has been previously reported as a low-penetrance mutation in CAPS, a finding in healthy populations and a factor that may exacerbate or modify the clinical phenotype of autoinflammatory disorders other than CAPS. An example of this is the case described by Singh-Grewal *et al*. [16] of a patient with *NLRP3 *V198M first seen at the age of 6 years with progressive sensorineural deafness followed within a couple of years by recurrent fever, abdominal pain, and arthritis. Screening for *MEFV *revealed she was a compound heterozygote for V726A, I692del, and E148Q, and was diagnosed with FMF. She was noncompliant to treatment with colchicine and died of renal AA amyloidosis at the age of 13 years. Another example, in this case of overlapping TRAPS/CAPS phenotype, was described in 2006 of a 36-year-old French woman and her mother, both with *NLRP3 *V198M and *TNFRSF1A *R92Q. Their symptoms, including fever, urticarial rash, arthralgia, myalgia, aphthosis, edema, fatigue, and conjunctivitis, were precipitated by exposure to heat and water. The proband's daughter, who only had *TNFRSF1A *R92Q, had a much milder disease, and the proband's grandmother and stepbrother, who carried *TNFRSF1A *R92Q and *NLRP3 *V198M, respectively, were completely asymptomatic [17].

Variants in other hereditary periodic fever syndromes such as in FMF E148Q and in TRAPS R92Q have also been described with a number of inflammatory disorders [36-39]. These low-penetrance mutations/polymorphisms have a relatively high prevalence in certain populations; R92Q is present in 2% of North Americans and Irish [36,37,40], and E148Q, in 20% of Asians [41]. Although they may have clinically significant proinflammatory effects in some cases, their implication in the disease cause remains dubious [36,37,40,42].

The finding of V198M in a patient with Schnitzler syndrome is particularly intriguing. Schnitzler syndrome is an adult-onset, apparently acquired disease, which clinically closely resembles CAPS. Fewer than 100 cases have been reported since it was first described in 1972 [43,44]. The main clinical features include fever, an urticarial rash, and muscle, bone, and/or joint pain. Other manifestations include lymphadenopathy (45%), hepatosplenomegaly (12%), and deafness (1%) [43]. A monoclonal IgM component is the biologic hallmark of the disease and is thought to be central to its pathogenesis, although whether and how this is linked to increased secretion of IL-1 has not been established. Treatment was very unsuccessful until the introduction of anti IL-1 therapies, which result in complete disease remission and support the central role of IL-1 in the disease process [43,44]. A recent report describes the *NLRP3 *V198M variant in a patient with Schnitzler syndrome [18]. Our finding of a second case supports the suggestion that a molecular link may exist between the NLRP3 inflammasome and the mechanism by which the IgM paraprotein induces inflammation.

This is the largest reported cohort of individuals with V198M assessed in a single center. Our findings that V198M was associated with a variety of CAPS and other autoinflammatory phenotypes in 12 cases and was identified in seven asymptomatic individuals emphasizes the complexity of the genetics of these syndromes. In our center, the allele frequency of V198M among patients with clinical evidence of fever syndrome, excluding the family screening, was 0.8%, twice that reported in European healthy controls [11]. The valine amino acid at position 198 is not conserved in evolution, suggesting that it does not have key role in the function of cryopyrin. The different phenotypes observed in association with V198M further suggest that other, currently unknown, genetic and environmental factors contribute to its involvement in autoinflammatory disease. It is possible that variants of other components of the NLRP3 inflammasome complex could play a role in disease pathogenesis, although this has not yet been systematically explored on a large scale.

## Conclusions

The *NLRP3 *V198M variant shows variable expressivity and reduced penetrance. It may be associated with classic inherited or apparently sporadic CAPS, and with atypical autoinflammatory disease of varying severity, intriguingly including Schnitzler syndrome. The factors that influence the pathogenic consequences of this variant remain unknown. However, the remarkable response to IL-1 blockade in all but one individual in our series confirms that the clinical features are indeed mediated by IL-1.

## Abbreviations

CAPS: cryopyrin-associated periodic syndrome; CINCA: chronic infantile neurologic cutaneous articular syndrome; CRP: C-reactive protein; FCAS: familial cold autoinflammatory syndrome; FMF: familial Mediterranean fever; HIDS: hyper-IgD and periodic fever syndrome; MKD: mevalonate kinase deficiency; MWS: Muckle-Wells syndrome; NOMID: neonatal-onset multisystem inflammatory disease; SAA: serum amyloid A protein; SAPHO: synovitis: acne: pustulosis: hyperostosis: and osteitis; SoJIA: systemic-onset juvenile idiopathic arthritis; TRAPS: TNF-receptor-associated periodic syndrome.

## Competing interests

The authors declare that they have no competing interests.

## Authors' contributions

The manuscript has been read and approved by all of the authors. The contribution of each author listed in this publication was as follows: study conception and design, and final approval of the manuscript was performed by HJL and PNH; drafting of the manuscript and data analysis was performed by DMR and HJL; genetic screening was performed by DMR, HT, TR, and AB; patient clinical assessment was performed by: TL, NMS, JDG, PW, BM, HJL, and PNH.
